# SARS-CoV-2 infection in public hospital medical doctors in an Eastern Cape metro

**DOI:** 10.4102/sajid.v37i1.335

**Published:** 2022-03-10

**Authors:** Ruan Spies, Matthew Potter, Sudarshan Govender, Luke Kirk, Simon Rauch, John Black

**Affiliations:** 1 Department of Medicine, Port Elizabeth Hospital Complex, Gqeberha, South Africa; 2 Department of Infectious Diseases, Livingstone Hospital, Gqeberha, South Africa; 3 Department of Medicine, Faculty of Health Sciences, University of Cape Town, Cape Town, South Africa

**Keywords:** SARS-CoV-2, COVID-19, occupational infection, occupational health, healthcare workers, infection prevention and control

## Abstract

**Background:**

Evidence-based Infection Prevention and Control (IPC) measures are critical in protecting medical doctors from severe acute respiratory syndrome coronavirus 2 (SARS-CoV-2) infection. Concerns surrounding access to personal protective equipment (PPE), compliance with IPC measures and the quality of available PPE have been raised as possible causes for high rates of SARS-CoV-2 infection in medical doctors in high transmission settings. This study aimed to determine the prevalence of SARS-CoV-2 infection and the risk factors for occupational infection in doctors in the hospitals in Nelson Mandela Bay (NMB).

**Methods:**

We conducted a cross-sectional study wherein we electronically surveyed medical doctors in public-sector NMB hospitals from 01 March 2020 to 31 December 2020. We collected demographic, health, occupational and SARS-CoV-2 infection and exposure data. Categorical data were described as proportions and a multiple variable logistic regression model was used to identify risk factors for SARS-CoV-2 infection.

**Results:**

The survey was distributed amongst 498 doctors, 141 (28%) of whom replied. Forty-three (31%) participants reported that they had tested positive for SARS-CoV-2 during the study period. Eighty-nine participants (64%) reported inadequate access to PPE whilst only 68 (49%) participants adhered to PPE recommendations when interacting with patients with confirmed or suspected SARS-CoV-2 infection. We were unable to identify any significant predictors of SARS-CoV-2 infection.

**Conclusion:**

This study demonstrates a high prevalence of SARS-CoV-2 infection in public hospital doctors in NMB. Most participants reported inadequate access to PPE and poor compliance with IPC protocols. These findings suggest an urgent need for the improved implementation of IPC measures to protect doctors from SARS-CoV-2 infection.

## Introduction

The novel coronavirus SARS-CoV-2 (severe acute respiratory syndrome coronavirus 2), which causes the acute respiratory illness coronavirus disease 2019 (COVID-19), emerged in Wuhan, China at the end of 2019 and spread rapidly across the globe.^1^ In South Africa (SA), Nelson Mandela Bay (NMB) in the Eastern Cape province became a ‘COVID-19 hotspot’ amassing a total of 51 262 cases and 2288 deaths by 31 December 2020, the period encompassing the first two waves of SARS-CoV-2 infection in the region.^2^ By the 1st of October 2021, following three waves of SARS-CoV-2 infection, 93 488 cases and 4082 deaths had been recorded. The pandemic put severe strain on both public and private healthcare services in the metro, attracting local and international media attention.^3,4,5^

Healthcare workers (HCWs) may be at greater risk of contracting SARS-CoV-2 than the general population with this increased risk likely explained by occupational exposure to patients with COVID-19.^6,7^ Furthermore, medical doctors are responsible for performing most aerosol generating procedures (AGPs), which are associated with increased risk of transmission of respiratory pathogens.^8^ These procedures include endotracheal intubation, manual ventilation, insertion of nasogastric tubes and collection of oropharyngeal and nasopharyngeal swabs for SARS-CoV-2 testing. The COVID-19 may result in mortality and long-term morbidity^9^ whilst SARS-CoV-2 infection may result in prolonged periods of workplace absenteeism as a result of quarantine and isolation, resulting in staff shortages. It is thus imperative that the risk of transmission amongst HCWs and within healthcare facilities is minimised. The National Institute of Communicable Diseases (NICDs) recommends infection prevention and control (IPC) measures such as regular handwashing with alcohol-based hand wash, the isolating and cohorting of patient with suspected and confirmed COVID-19, the use of personal protective equipment (PPE) for HCWs and the safe donning and doffing of PPE by HCWs.^10^ Personal protective equipment recommendations for standard precautions include the use of non-sterile gloves, surgical masks, aprons or gowns and goggles or face shields.^10^ For AGPs, the use of N95 respirators is recommended, replacing surgical masks.^10^ These respirators have been shown to reduce the risk of SARS-CoV-2 infection in HCWs but because of global shortages are not widely available across Africa.^8,11^ Furthermore, KN95 respirators that have been widely distributed to HCWs across SA have been demonstrated to fail the stipulated safety thresholds associated with the protection of HCWs.^12^

We predict a high prevalence of SARS-CoV-2 infection in public hospital medical doctors in NMB because of the high prevalence of SARS-CoV-2 infection in the metro, the performing of AGPs by doctors and the lack of access to N95 respirators.

This study aimed to describe the prevalence of SARS-CoV-2 infection in medical doctors working in the public hospitals NMB and to identify occupational factors associated with SARS-CoV-2 infection.

## Methods

### Study design and setting

This cross-sectional study took place from 19 May to 31 May 2021. All medical doctors working at Livingstone Hospital, Dora Nginza Hospital, Port Elizabeth Provincial Hospital and Uitenhage Provincial Hospital between 1 March and 31 December 2020 were invited to participate in the study. This period was selected as it encompassed the start of the ‘first wave’ and most of the ‘second wave’ of SARS-CoV-2 infections in NMB, whilst also avoiding the inclusion of new doctors starting in the public hospitals of NMB on 01 January 2021.

Participation involved the completion of an online questionnaire, administered via an online survey tool (Google Forms) and distributed through link-enabled platforms including email and WhatsApp. The questionnaire was distributed to individual email addresses and cell phone numbers. No incentives were offered for participation. Participants’ responses were anonymous and they were only able to submit the completed questionnaire once.

The questionnaire was developed by the study authors and was piloted in a group of 10 public-sector doctors based outside NMB, with revisions subsequently made based on their feedback. The questionnaire included demographic, health and occupational information and information related to SARS-CoV-2 exposure and infection.

### Ethical considerations

Research approval was granted by the University of Cape Town Human Research Ethics Committee (HREC 155/2021), the Eastern Cape Department of Health Research Committee and hospital management.

### Data analysis

All data were stored in password-protected spreadsheets and analysis was performed using R (RStudio, Inc). Categorical variables were described as proportions whilst continuous variables were described with medians and interquartile ranges. Continuous variables were compared between groups using the Wilcoxon rank-sum test whilst categorical variables were compared between groups using the Pearson’s chi-squared test. A *p*-value of < 0.05 was considered to represent a statistically significant difference between groups. Simple logistic regression was used to describe the magnitude of association of predictor variables and the outcome variable (self-reported SARS-CoV-2 infection as determined by a polymerase chain reaction [PCR] test performed on a nasopharyngeal or oropharyngeal swab). The variable ‘Rank’ was dichotomised with junior = intern and senior = medical officer, registrar or consultant. The variable ‘Adequate PPE’ was defined as ready availability of PPE as per NICD guidelines.^10^ This included access to non-sterile or sterile gloves, surgical masks or KN95 respirators, protective gowns or disposable aprons, face-shields or eye goggles and N95 respirators or KN95 respirators. Predictor variables associated with the outcome with p < 0.1 were included in a multiple variable logistic regression model, which also contained predictor variables previously identified in the literature as risk factors for SARS-CoV-2 infection. These include inadequate PPE, performing AGPs and high-risk exposure to an individual with confirmed SARS-CoV-2.^13,14,15^ Results were reported with 95% confidence intervals and *p* < 0.05 was considered as significant.

## Results

**FIGURE 1:**
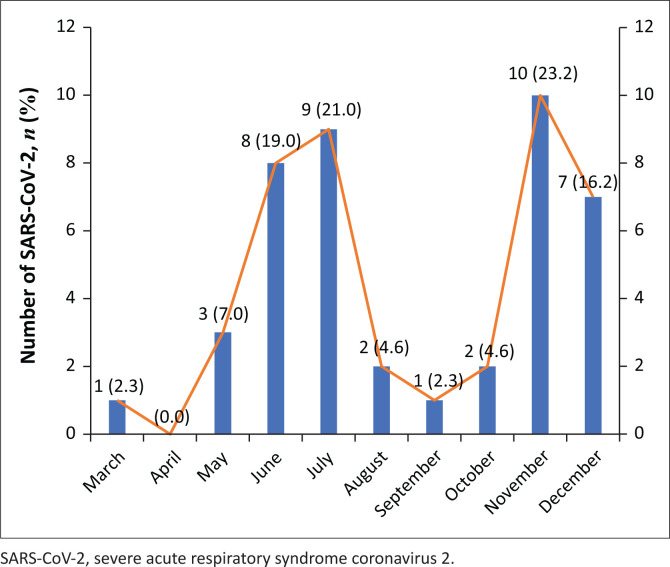
Month during which SARS-CoV-2 infections occurred in surveyed medical doctors at Nelson Mandela Bay Public Hospitals, March 2020 – December 2020.

The questionnaire was distributed amongst 498 doctors across four hospitals and 22 departments, 141 (28.3%) of whom responded. Two participants were excluded as they were not employed in the public hospitals of NMB between 01 March and 31 December 2020. The characteristics of the participants are described in Table 1. A total of 30.9% (43/139) of participants reported that they had tested positive for SARS-CoV-2 between 01 March and 31 December 2020. The majority of participants who tested positive for SARS-CoV-2 missed 10–14 days of work because of the infection. Internal medicine represented the domain during which the greatest number of infections occurred (30%) whilst the majority of infections occurred in November 2020 and December 2020 (40%) (Table 2) (Figure 1).

**TABLE 1:** Demographic characteristics of surveyed Nelson Mandela Bay Public Hospital medical doctors.

Characteristic	Overall				SARS-CoV-2 positive				SARS-CoV-2 negative				*p*
	*n*	%	Median	IQR	*n*	%	Median	IQR	*n*	%	Median	IQR	
**Number of participants**													
Age	139	-	32	26–35	43	30.9	27	25–35	96	69.1	28	26–35	0.29
**Gender**													0.42
Female	75	54.0	-	-	21	48.8	-	-	54	56.3	-	-	-
Male	64	46.0	-	-	22	51.2	-	-	42	43.7	-	-	-
**Rank**			-	-			-	-			-	-	0.07
Junior (Intern)	68	48.9	-	-	26	60.5	-	-	42	43.7	-	-	-
Senior (Medical officer/Registrar/Consultant)	71	51.1	-	-	17	39.5	-	-	54	56.3	-	-	-
**Number of comorbidities**													0.56
0	93	66.9	-	-	28	65.1	-	-	65	67.7	-	-	-
1	31	22.3	-	-	10	23.3	-	-	21	21.9	-	-	-
2	12	8.6	-	-	5	11.6	-	-	7	7.3	-	-	-
3	3	2.2	-	-	0	-	-	-	3	3.1	-	-	-

SARS-CoV-2, severe acute respiratory syndrome coronavirus 2; IQR, interquartile range.

**TABLE 2:** SARS-CoV-2 exposure and infection.

Variable	*n*	%
SARS-CoV-2 positive	43	30.9
High-risk SARS-CoV-2 exposure requiring isolation	75	54.0
Hospitalised due to COVID-19	2	4.7
**Days of work missed because of high-risk SARS-CoV-2 exposure**		
0	10	13.3
1–4	34	45.3
5–9	13	17.3
10–14	14	18.7
15 or more	4	5.3
**Days of work missed due to SARS-CoV-2 infection**		
1–4	1	2.3
5–9	14	32.3
10–14	21	48.8
15 or more	7	16.3
**Department in which SARS-CoV-2 infection occurred**		
Internal medicine	13	30.2
Obstetrics and gynaecology	8	18.6
Emergency department/family medicine	7	16.3
General surgery	4	9.3
Orthopaedic surgery	4	9.3
Urology	2	4.7
Oncology	1	2.3
Anaesthetics	1	2.3
Cardiothoracic surgery	1	2.3
Intensive care unit	1	2.3
Paediatrics	1	2.3

SARS-CoV-2, severe acute respiratory syndrome coronavirus 2.

**FIGURE 2:**
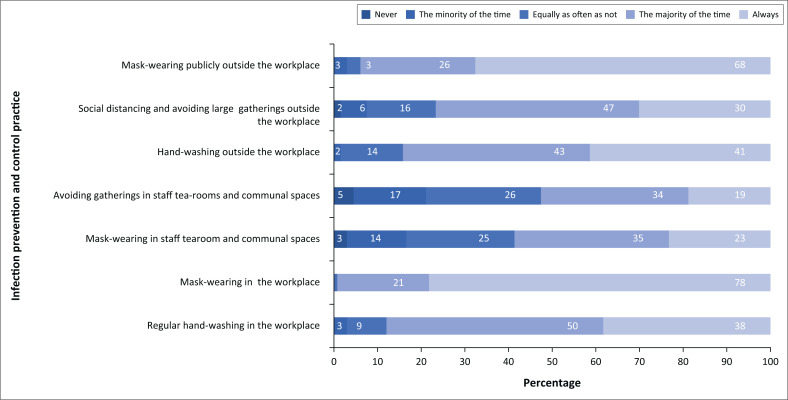
Infection prevention and control behaviours of surveyed doctors at Nelson Mandela Bay Public Hospitals.

The majority of participants, 113 (81.3%) reported to have received training on the use of PPE. Eighty nine participants (64%) reported inadequate access to PPE when compared with NICD guidelines whilst only 68 (48.9%) participants reported always wearing standard-precaution PPE when exposed to patients with confirmed or suspected SARS-CoV-2 infection. A total of 117 participants (84.2%) performed AGPs, however only 56 (47.5%) of these participants performed AGPs whilst wearing the recommend PPE. A total of 84 participants (60.4%) reported that there were no workplace protocols for social distancing in staff common areas including staff tearooms and theatres (Table 3). Participants’ compliance with IPC measures, both inside and outside the workplace, is displayed in Figure 2. Most participants reported that they wore masks, avoided large gatherings, adhered to social distancing and regularly washed their hands in the workplace most of the time. Only 53% of the surveyed participants avoided gatherings in staff tea-rooms and communal workspaces most of the time and only 58% of surveyed participants wore mask in staff tea-rooms and communal spaces most of the time.

**TABLE 3:** SARS-CoV-2 infection possible risk factors.

COVID-19 risk factor questions	Overall (*n* = 139)		SARS-CoV-2 positive (*n* = 43)		SARS-CoV-2 negative (*n* = 96)		*p*
	*n*	%	*n*	%	*n*	%	
Inadequate access to alcohol-based handwash	28	20.1	13	30.2	15	15.6	0.05
Inadequate access to PPE as per NICD recommendations	89	64.0	27	62.8	62	64.6	0.84
Workplace social distancing protocols	55	39.6	17	39.5	38	39.6	0.99
Received training on use of PPE	113	81.3	37	86.0	76	79.2	0.34
Regular workplace symptom screening	79	56.8	26	60.5	53	55.2	0.56
Appropriate use of standard precaution PPE when exposed to patients with suspected or confirmed SARS-CoV-2 infection	68	48.9	24	55.8	44	45.8	0.28
Performed aerosol generating procedures (AGPs)	117	84.2	37	86.0	80	83.3	0.69
Appropriate use of PPE when performing AGPs	56	40.3	16	37.2	40	41.7	0.54
High-risk SARS-CoV-2 exposure requiring isolation	75	54.0	25	58.1	50	52.1	0.51

SARS-CoV-2, severe acute respiratory syndrome coronavirus 2; NICD, National Institute of Communicable Diseases; PPE, personal protective equipment.

Junior rank and poor access to alcohol-based handwash were associated with self-reported SARS-CoV-2 infection following univariate logistic regression with odds ratios 1.97 (95% confidence interval [CI]: 0.95–4.10, *p* = 0.07) and 2.34 (95% CI: 1.00–5.49, *p* = 0.05), respectively. Junior rank and poor access to alcohol-based handwash were then included in a multiple variable logistic regression model, which also included the predictors inadequate PPE, performing AGPs and high-risk exposure to individuals with confirmed SARS-CoV-2 infection. This model failed to identify any statistically significant predictors of SARS-CoV-2 infection (Table 4).

**TABLE 4:** Multiple variable logistic regression: Risk factors for SARS-CoV-2 infection.

Variable	OR	95% CI	*p*
Junior rank	1.76	0.80–3.86	0.16
Inadequate personal protective equipment (PPE)	0.74	0.33–1.65	0.46
Lack of access to alcohol-based handwash	2.20	0.88–5.50	0.09
Performed aerosol generating procedures (AGPs)	0.93	0.32–2.73	0.89
High-risk SARS-CoV-2 exposure requiring isolation	1.05	0.49–2.24	0.90

SARS-CoV-2, severe acute respiratory syndrome coronavirus 2; OR, Odds ratio; CI, confidence interval.

## Discussion

In this study we report a high prevalence of self-reported SARS-CoV-2 infection in medical doctors working in the public hospitals of NMB between 01 March 2020 and 31 December 2020, the period comprising the first two waves of SARS-CoV-2 infection in the region. Additional significant findings include low rate of access to appropriate PPE and low rates of correct PPE usage.

Almost one-third of the participants in our study reported a positive SARS-CoV-2 PCR test between 01 March 2020 and 31 December 2020. The true number of infections is possibly even higher considering an estimated one-third SARS-CoV-2 infections are asymptomatic and therefore unlikely to have been detected and reported.^16^ Estimates of infection in HCWs worldwide vary widely across period, setting and diagnostic modality. Surveillance data from SA, monitoring infection in HCWs employed by Anova Health Institute, demonstrated that 14% of employees tested positive for SARS-CoV-2 infection by PCR by the end of September 2020.^17^ A total of 34.6% of the participants in a cohort study of HCWs at Chris Hani Baragwanath tested positive for SARS-CoV-2 by PCR between April and September 2020^18^ whilst seroprevalence data from HCWs at a Cape Town paediatric hospital, from 1st May to mid-July 2020, suggested 10.4% of participants had been infected with SARS-CoV-2.^19^ A systematic review of 49 studies and 127 480 HCWs across Europe, North America, Asia and Africa estimated an overall seroprevalence of 8.7% by the end of August 2020^20^ whilst data from India described a seroprevalence of 25.6% in HCWs as of January 2021.^21^ Studies that did not include data from the latter parts of 2020 may have missed large numbers of infections comprising the ‘second wave’ of SARS-CoV-2 infections. In this study, as many infections were reported during November 2020 and December 2020 – the period that accounted for the bulk of the ‘second wave’ of infections in NMB – as in the June–July 2020 ‘first wave’ of infections.

It is unsurprising that the majority of SARS-CoV-2 infections in our study occurred in the domains of Internal Medicine and Family Medicine (including the Emergency Department) as doctors in these domains are likely to have been exposed to greater numbers of patients with COVID-19 than doctors in other domains. The study from Chris Hani Baragwanath Hospital also describes higher infection rates in the domain of Internal Medicine, however, unlike our study, 61.7% of HCWs in internal medicine were infected with SARS-CoV-2.^18^ Unexpectedly, nearly 20.0% of infections occurred in obstetrics and gynaecology, a domain with less contact to patients with severe COVID-19. It is unclear whether these infections were related to a clustered workplace outbreak or poor IPC practices with exposure to patients with asymptomatic infection. Another possible explanation for this observation may be the disruptions to elective surgical admissions during the pandemic, and subsequently lower patient volumes in surgical disciplines, which obstetrics would have been less affected by, maintaining expected patient volumes.

Several comorbidities including obesity, diabetes mellitus, cardiac disease, chronic kidney disease and chronic pulmonary disease have been identified as risk factors for severe COVID-19.^22^ In our study, one-third of the participants reported at least one comorbidity, with 10.0% reporting two or more comorbidities. Two participants in our study (4.7% of those with SARS-CoV-2 infection) were admitted to hospital with COVID-19. In a letter to parliament, health minister Dr Zweli Mkize reported that a total of 339 public-sector HCWs had died because of COVID-19 between March and November 2020.^23^ A meta-analysis on SARS-CoV-2 infection in HCWs, including studies conducted until July 2020, demonstrated a 15.0% prevalence of hospitalisation and a 1.5% prevalence of death.^24^ Although difficult to quantify, the death of any public-sector medical doctor is likely to compromise healthcare service delivery owing to the loss of experience in an already overburdened, under-resourced setting.

A concerning low proportion of participants adhered to recommendations on the appropriate use of PPE when interacting with patients with suspected or confirmed SARS-CoV-2 infection. This is despite the fact that most participants reported receiving training on the use of PPE. Indeed, a 2021 study from Ghana demonstrated far higher rates of PPE compliance (90.6%) than identified in our study.^25^ Although adequate access to PPE was not associated with SARS-CoV-2 infection in our study, the use of appropriate PPE has been consistently associated with decreased risk of SARS-CoV-2 infection in HCWs.^13,14,26^ Our study did not investigate reasons for poor adherence to PPE recommendations, however a systematic review by Alhumaid et al. identified three major factors associated with IPC compliance, namely knowledge, education and training and experience.^27^ It may be that the PPE training provided to our participants did not emphasise the benefits of PPE use and the risks of not complying with PPE recommendations. Personal protective equipment training sessions were generally once-off, stand-alone sessions and it may be that a more longitudinal training programme with more frequent reinforcement might result in better compliance. The factors associated with non-compliance with IPC measures in the Alhumaid et al. systematic review included high workloads and time constraints^27^ – issues, which existed in the SA public healthcare sector prior to the pandemic and which have been greatly exaggerated during the pandemic. Infection prevention and control ‘fatigue’ – the decreased compliance with IPC measures over time as HCWs get tired under the working conditions forced by the pandemic may also contribute to poor adherence to PPE recommendations. There is unfortunately a paucity of research on this phenomenon and our study did not compare PPE adherence during the first and second waves of SARS-CoV-2 infections. Another factor contributing to poor compliance with PPE recommendations may be inadequate access to appropriate PPE, reported by most participants in our study. Poor access to appropriate PPE has been a global concern throughout the pandemic, disproportionately affecting poorer countries.^11^ Similar to our findings, 70% of HCWs reported a lack of access to PPE in a study conducted in Brazil, Colombia and Ecuador.^28^ Concerns regarding access to PPE for HCWs in SA have been present since the beginning of the pandemic, with novel approaches to PPE preservation including decontamination of used PPE required.^29^ Unfortunately, the procurement of PPE for HCWs in SA has been marred by allegations of corruption and financial irregularities, with frontline HCWs left to manage the consequences.^28^

Participants reported good compliance with other IPC measures including handwashing, wearing of mask and avoiding gatherings, both inside and outside the workplace. The prevalence of SARS-CoV-2 infection remained high despite these measures. Participants’ response to these questions were inherently subjective and vulnerable to recall bias. Furthermore, our study did not investigate environmental factors related to IPC such as patient overcrowding, the cohorting and transfer of patients under investigation for SARS-CoV-2 infection or waste management and workplace hygiene and cleaning – another well publicised crisis within NMB public hospitals.^30^ These factors may all have influenced SARS-CoV-2 infection prevalence. Future work should include broader and more objective evaluations of hospital IPC programmes and their relationship with SARS-CoV-2 infection.

Our study has several limitations including a relatively small sample size and low questionnaire response rate. These factors reduce the representativeness of our study and may explain why we were unable to identify any significant predictors of SARS-CoV-2 infection. Our study may be susceptible to selection bias because of the non-random, voluntary inclusion of participants. It is however unclear which participant characteristics may have been selected for by this sampling method. Our outcome measure, self-reported SARS-CoV-2 infection defined by a positive PCR test, is not independently verifiable, however, self-reported data may have an advantage over incomplete surveillance data. We were unable to identify the temporal relationship between possible predictors and our outcome measure because of the cross-sectional design of the study. Furthermore, our study was unable to ascertain the site and source of SARS-CoV-2 infection, which may have occurred within or outside the workplace and the additional health system factors that may have contributed to infections. An additional limitation of our study is its temporal setting as it only describes the first two waves of SARS-CoV-2 infection in the region. The COVID-19 pandemic is rapidly evolving with epidemiological data evolving accordingly. More recent studies on SARS-CoV-2 infection in HCWs are necessary and future studies should include seroprevalence data in order to attain a broader understanding of SARS-CoV-2 infection in SA doctors. Vaccination of SA HCWs commenced in February 2021 through the Sisonke trial using the Ad.26.COV2.S (Johnson and Johnson) vaccine, which has been demonstrated to offer protection against symptomatic and severe COVID-19.^31^ It is expected that vaccination in HCWs will dramatically reduce the incidence of SARS-CoV-2 infection in this group. Although data from the Sisonke trial are yet to be published, early data are very promising, suggesting 65% – 66% prevention of hospitalisation and 91% – 95% prevention of death.^32^ Several international studies have already demonstrated the efficacy of vaccines in reducing both symptomatic and asymptomatic SARS-CoV-2 infection in HCWs.^33,34^

## Conclusion

Our study demonstrates a high prevalence of SARS-CoV-2 infection in public hospital medical doctors in NMB between 01 March 2020 and 31 December 2020. Poor access to PPE and poor compliance with PPE recommendations was reported by most participants. We were unable to identify any risk factors associated with SARS-CoV-2 infection. Our findings suggest an urgent need for the implementation of measures to protect doctors from SARS-CoV-2 infection including widespread vaccination and appropriate IPC measures.
